# *Enterococcus durans* TN-3 Induces Regulatory T Cells and Suppresses the Development of Dextran Sulfate Sodium (DSS)-Induced Experimental Colitis

**DOI:** 10.1371/journal.pone.0159705

**Published:** 2016-07-20

**Authors:** Toshihiro Kanda, Atsushi Nishida, Masashi Ohno, Hirotsugu Imaeda, Takashi Shimada, Osamu Inatomi, Shigeki Bamba, Mitsushige Sugimoto, Akira Andoh

**Affiliations:** 1 Department of Medicine, Shiga University of Medical Science, Seta-Tsukinowa, Otsu, Shiga, Japan; 2 Central Research Laboratories, Nichinichi Pharmaceutical Corporation Ltd., Tominaga, Iga, Mie, Japan; University of South Carolina School of Medicine, UNITED STATES

## Abstract

**Background and Aims:**

Probiotic properties of *Enterococcus* strains have been reported previously. In this study, we investigated the effects of *Enterococcus (E*.*) durans* TN-3 on the development of dextran sulfate sodium (DSS) colitis.

**Methods:**

BALB/c mice were fed with 4.0% DSS in normal chow. Administration of TN-3 (10mg/day) was initiated 7days before the start of DSS feeding. Mucosal cytokine expression was analyzed by real time-PCR and immunohistochemistry. The lymphocyte subpopulation were analyzed by flow cytometry. The gut microbiota profile was analyzed by a terminal-restriction fragment length polymorphism method (T-RFLP).

**Results:**

The disease activity index and histological colitis score were significantly lower in the DSS plus TN-3 group than in the DSS group. The mucosal mRNA expression of proinflammatory cytokines (IL-1β, IL-6, IL-17A and IFN-γ) decreased significantly in the DSS plus TN-3 group as compared to the DSS group. The proportion of regulatory T cells (Treg cells) in the mucosa increased significantly in the DSS plus TN-3 group as compared to the DSS group. Both fecal butyrate levels and the diversity of fecal microbial community were significantly higher in the TN-3 plus DSS group than in the DSS group.

**Conclusions:**

*E*. *durans* TN-3 exerted an inhibitory effect on the development of DSS colitis. This action might be mediated by the induction of Treg cells and the restoration of the diversity of the gut microbiota.

## Introduction

Inflammatory bowel disease (IBD), such as ulcerative colitis (UC) and Crohn’s disease (CD), are chronic intestinal inflammatory disorders of unknown etiology [1, 2]. Recent studies suggested that a dysfunction of the host immune response against dietary factors and the commensal bacteria plays an important role in the pathogenesis of IBD [3–7].

Probiotics are live microorganisms that provide beneficial effects to the host when administered in adequate amounts [8]. They exert these effects by modulating the gut microbiota and promoting mucosal barrier functions and resistance to pathogens. Now, probiotics are considered to be a therapeutic option for inducing or maintaining clinical remission of IBD.

Most of probiotics consist of *Lactobacillus* spp., *Bifidobacterium* spp. and *Enterococcu*s spp. [9]. Among *Enterococcus* spp., *Enterococcus durans* (*E*. *durans*) has been reported to exert various probiotic effects. For example, *E*. *durans* strain 6HL, isolated from the vagina of heathy women, possesses the ability to inhibit the growth of pathogenic microorganisms [10]. Avram-Hananel *et al*. reported that *E*. *durans* strain M4-5, isolated from the human colon, improved intestinal inflammation [11]. Kondoh et al. reported that *E*. *durans* strain TN-3, isolated from deep seawater, effectively suppressed dermal eosinophil accumulation in allergen-primed mice [12].

Several animal models of experimental colitis induced by chemical agents have been employed to investigate the pathophysiology of IBD [13, 14]. DSS colitis model is one of the widely used models owing to the reproducibility. DSS colitis is morphologically characterized by epithelial cell damage, ulceration, submucosa edema, and the infiltration of granulocytes and mononuclear immune cells. Therefore, DSS colitis model is considered to exhibit features of relevance of human ulcerative colitis [15–17]. In this study, we used the regimen of a continuous administration of DSS to examine the effect of preventive treatment of TN-3 on the induction of acute phase of intestinal injury.

Here, we investigated the effects of *E*. *durans* TN-3 on dextran sulfate sodium (DSS)-induced colitis to explore its therapeutic potential for IBD patients. We further analyzed the effects of *E*. *durans* TN-3 on the mucosal lymphocyte subpopulation and fecal levels of short-chain fatty acids.

## Materials and Methods

### Experimental animals and induction of colitis

BALB/cAJcl mice (Six to eight week-old females) were purchased from CLEA Japan (Tokyo, Japan). They were acclimatized for one week before the experiment, and were housed individually in a room maintained at 22°C under a 12-h day/night cycle throughout the experiments. They were allowed free access to rodent chow (MF; Oriental Yeast Co., Ltd, Tokyo, Japan) and drinking water. Experimental colitis was initiated by the oral administration of 4% DSS (molecular weight 5000; Wako Pure Chemical Industries, Ltd, Osaka, Japan) mixed with normal chow. Mice were divided into 4 groups; control mice, TN-3-treated mice, 4% DSS-treated mice, and 4% DSS plus TN-3-treated mice. For the examination of preventive effect of TN-3 on the colitis, TN-3 (10mg/day in 0.3ml phosphate buffered saline (PBS)) was administrated by oral gavage 7 days before the start of DSS administration. For the examination of the therapeutic effect of TN-3 on the colitis, the administration of TN-3 (10mg/day in 0.3ml PBS) started simultaneously with starting of DSS. The mice were euthanized at day12 under diethyl ether anesthesia by quick cervical distortion to minimize animal suffering. This study was carried out in strict accordance with the recommendations in the Guide for the Care and Use of Laboratory Animals of the National Institutes of Health. This study protocol was approved by the Animal Care and Use Committee of Shiga University of Medical Science (Otsu, Japan) (Permit number:2013-9-8).

### Preparation of *E*. *durans* TN-3

*E*. *durans* strain TN-3 was isolated from deep-sea water in Toyama bay. TN-3 was cultures for 18h at 30°C in a broth medium containing 2.46% (w/v) glucose, 1.4% (w/v) yeast extract, 0.77% (w/v) peptone and 4.39% (w/v) K_2_HPO_4_. After cultivation, the cells were collected by centrifugation and washed with distilled water. Heat-killed TN-3 was treated by autoclave for 10 min at 110°C and then lyophilized.

### Assessment of inflammation in DSS-induced colitis

Mucosal inflammation was assessed using the disease activity index (DAI) described previously [18]. Histologic evaluations were performed in a blinded fashion using a validated scoring system [19].

### Real-time polymerase chain reaction (real-time PCR)

The mRNA expression in the samples was assessed by real-time-polymerase chain reaction (PCR) analyses using a Light Cycler 480 system (Roche Applied Science, Tokyo, Japan) and SYBR Premix Ex Taq II (TAKARA, Otsu, Japan). The data were normalized versus β-actin mRNA. The oligonucleotide primers used in this study are shown in Table 1.

**Table 1:** PCR primers used in this study.

Gene	Accession number	Primers	
IL-1β	NM_008361	sense	5’-CAGGATGAGGACATGAGCACC-3’
		anti-sense	5’-CTCTGCAGACTCAAACTCCAC-3’
IL-6	NM_031168	sense	5’-GACAAAGCCAGAGTCCTTCAGAGA-3’
		anti-sense	5’-CTAGGTTTGCCGAGTAGATCTC-3’
IL-10	NM_010548	sense	5’-GTGAAGACTTTCTTTGAAACAAAG-3’
		anti-sense	5’-CTGCTCCACTGCCTTGCTCTTATT-3’
IL-17A	NM_010552	sense	5’-TCTCTGATGCTGTTGCTGCT-3’
		anti-sense	5’-CGTGGAACGGTTGAGGTAGT-3’
IFN-γ	NM_008337	sense	5’-TACTGCCACGGCAGTCATTGAA-3’
		anti-sense	5’-GCAGCGACTCCTTTTCCGCTTCCT-3’
β-actin	NM_007393	sense	5’-GTGGGCCGCCCTAGGCACCA-3’
		anti-sense	5’-CGGTTGGCCTTAGGGTTCAGGGGGG-3’

### Immunohistochemistry

Immunohistochemical analyses were performed according to a method described in our previous report [20]. Briefly, rabbit anti-IL-1β (clone H-153; Santa Cruz biotechnology Inc., Dallas, TX), goat anti-IL-6 (clone H-19; Santa Cruz Biotechnology Inc.), rat-anti-IFN-γ (clone XMG1.2; BioLegend, San Diego, CA), rabbit anti-IL-17A (clone H-132; Santa Cruz Biotechnology Inc.), and goat anti-IL-10 antibody (clone M-18; Santa Cruz Biotechnology Inc.) were used as the primary antibodies. After incubation with the primary antibodies, the sections were treated with HRP (horseradish peroxidase)-labeled anti-rabbit IgG, anti-goat IgG, or anti-rat IgG antibodies. Diaminobenzidine was used as a substrate for color development.

### Cell isolation and flow cytometry

Mononuclear cells were isolated from the lamina propria of the colon. The isolated cells were stained with PE-labeled anti-CD4 (clone RM4-4; eBioscience, San Diego, CA), Alexa Fluor 488-labeled anti-Foxp3 (clone MF-14; BioLgend), PECy7-labeled anti-F4/80 (clone BM8; eBioscience), and APC-labeled anti-Gr-1 antibody (clone RB6-8C5; eBioscience). The cells were analyzed using FACS Calibur (BD Biosciences, Franklin Lake, NJ) according to the methods described previously [21, 22].

### High-performance liquid chromatography

High-performance liquid chromatography (HPLC) was carried out for the analysis of stool extracts as previously described [23]. HPLC was performed using an Agilent 1120 Compact LC system (Santa Clara, CA) and a COSMOSIL 4.6×150mm 5C_18_-AR-Ⅱ column (nacalai tesque inc., Kyoto, Japan).

### DNA extraction and terminal restriction fragment length polymorphism (T-RFLP) analysis

DNA samples from feces were isolated using the method described previously [24]. The final concentration of DNA sample was adjusted to 10 ng/μl. T-RFLP analysis of the gut microbiota was performed according to the method described previously [24]. The T-RF fragments were divided into 30 operational taxonomic units (OTUs) as described by Nagashima et al. [25]. The prediction of bacteria was performed according to the *Bsl*I-digested T-RFLP database [25]. The diversity among the different samples was compared by the Shannon diversity index (SDI) [26, 27].

### Statistical analysis

The statistical significance of the differences was determined by Mann-Whitney U test. Differences resulting in *P* values less than 0.05 were considered to be statistically significant.

## Results

### Effects of TN-3 on the development of DSS colitis

**Fig 1:**
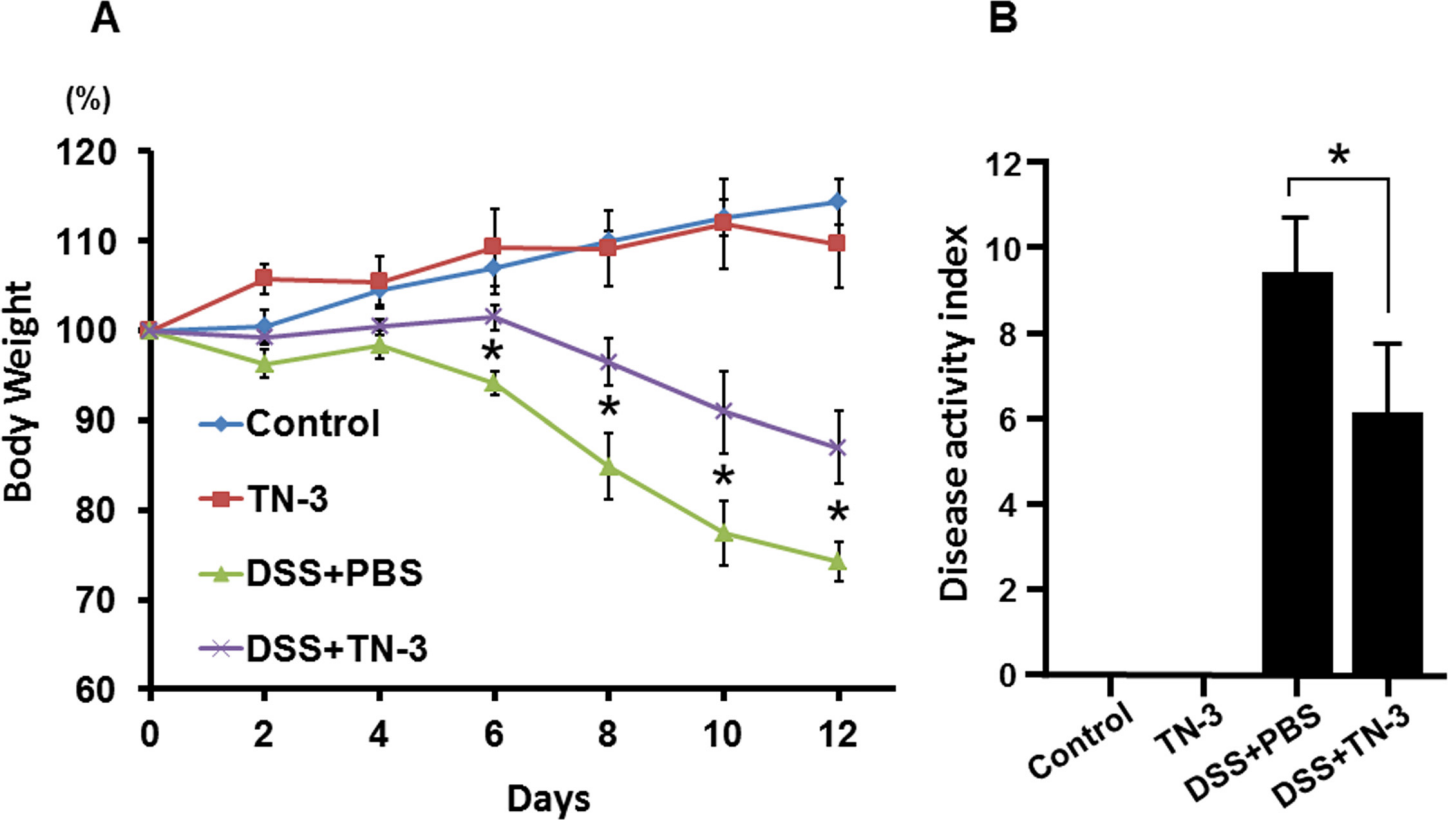
Effect of *E*.*durans* TN-3 on the development of DSS colitis. BALB/cAJcl mice were orally inoculated with *E*. *durans* TN-3 (10mg/day) for 7 days prior to the start of 4% DSS treatment. The mice were sacrificed at day12 for the experiments. (A) Changes in body weight. (B) Disease activity index on day 12. Data are expressed as means ± SD (n = 10 mice/group).*P < 0.05 between the DSS mice and the DSS plus TN-3 mice.

**Fig 2:**
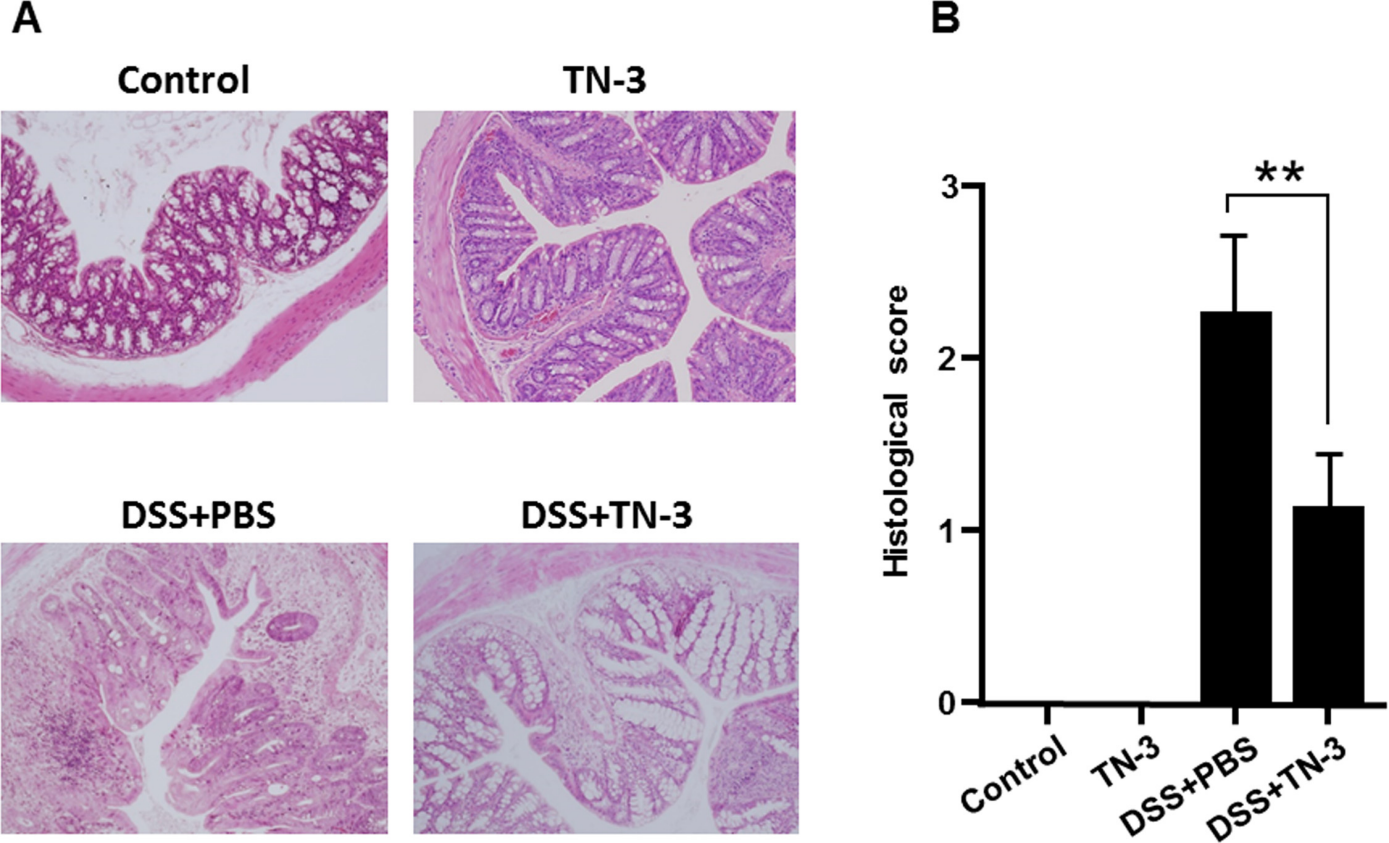
Histological evaluation of colitis. (A) Histologic findings of the colonic tissue on day 12. Hematoxylin and eosin staining. (original magnification x100). (B) Histological score. Data are expressed as means ± SD (n = 10 mice/group). *P < 0.05, **P < 0.01, n.s.: not significant.

**Fig 3:**
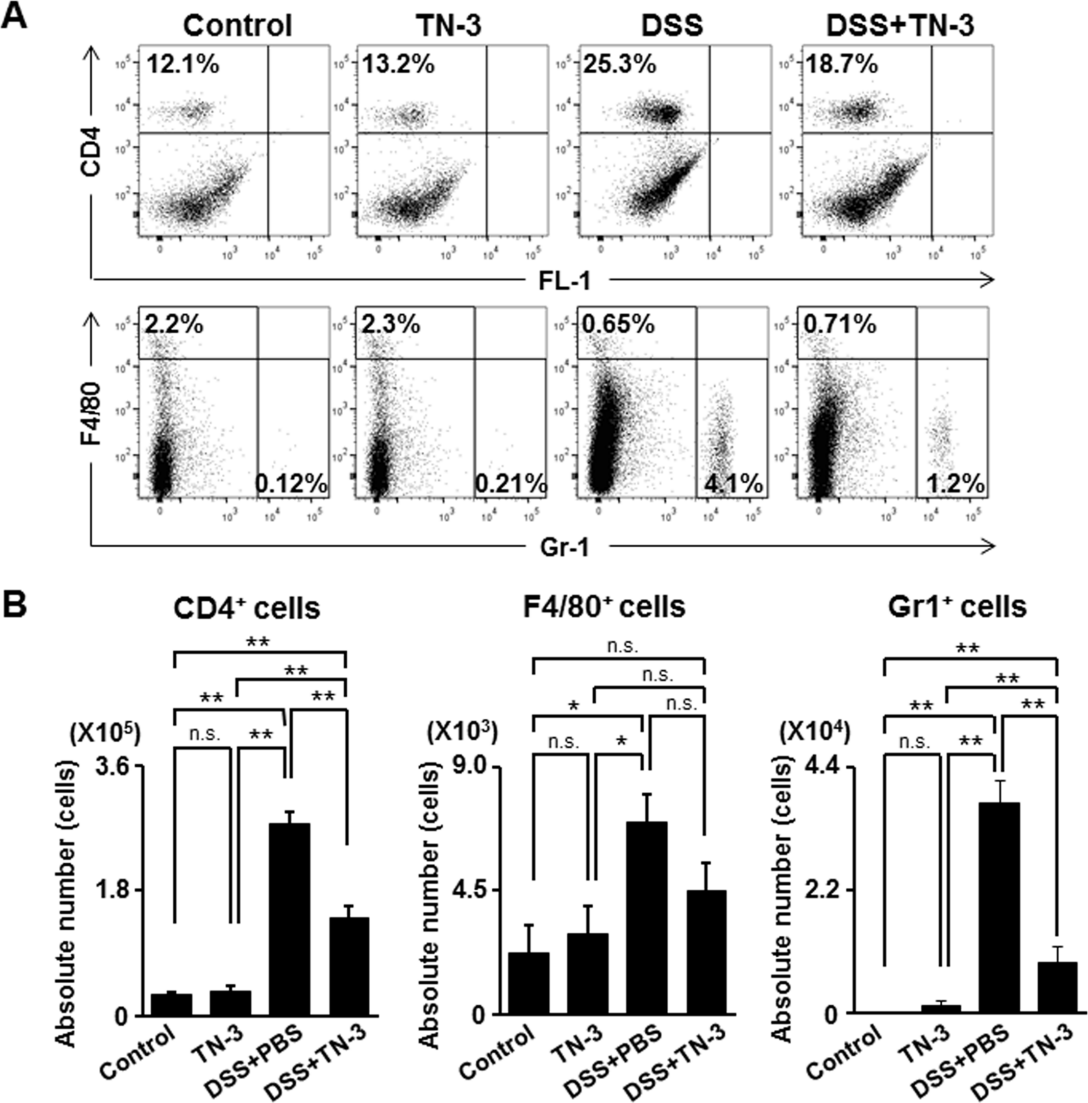
The proportion of the immune cells in the DSS colitis. (A) The Flow cytometric analysis for CD4^+^ T cells, F4/80^+^ macrophages, and Gr-1^+^ neutrophils in the colonic lamina propria. Data are representative of five independent experiments. (B) The absolute number of T cells, macrophages, and neutrophils in the colonic lamina propria. Data are expressed as means ± SD (n = 5 mice/group). *P < 0.05, **P < 0.01, n.s.: not significant.

To evaluate the preventive effects of TN-3 on the development of DSS colitis, we treated mice with TN-3 for 7 days prior to the start of DSS administration. As shown in Fig 1A, body weight (BW) was significantly lower in the DSS mice as compared to the DSS plus TN-3 mice. The disease activity index (DAI) was significantly higher in the DSS mice than the DSS plus TN-3 mice (Fig 1B). Furthermore, the histological inflammatory score was significantly lower in the DSS plus TN-3 mice than in the DSS mice (Fig 2A and 2B). The infiltration of immune cells also reflects the severity of colitis. Flow cytometric analysis for CD4^+^ T cells, F4/80^+^ macrophages, and Gr-1^+^ neutrophils in the colonic lamina propria was performed. As shown in Fig 3A and 3B, the infiltration of CD4^+^ T cells and Gr-1^+^ neutrophils was significantly suppressed in the DSS plus TN-3 mice as compared to the DSS mice. The infiltration of F4/80^+^ macrophages in the DSS plus TN-3 was also suppressed as compared to the DSS mice, but there was no significant difference between two groups.

We also examined the therapeutic effect of TN-3 on the development of colitis. We started the treatment of TN-3 at the same time of the starting of DSS administration. As shown in S1 Fig, BW in the TN-3 plus DSS mice was as low as the DSS mice, and the DAI in TN-3 plus DSS mice as high as the DSS mice. These results suggested that TN-3 has the preventive effect, but not the therapeutic effect, on the development of colitis.

### Effect of TN-3 on the mucosal mRNA expression of proinflammatory cytokines

**Fig 4:**
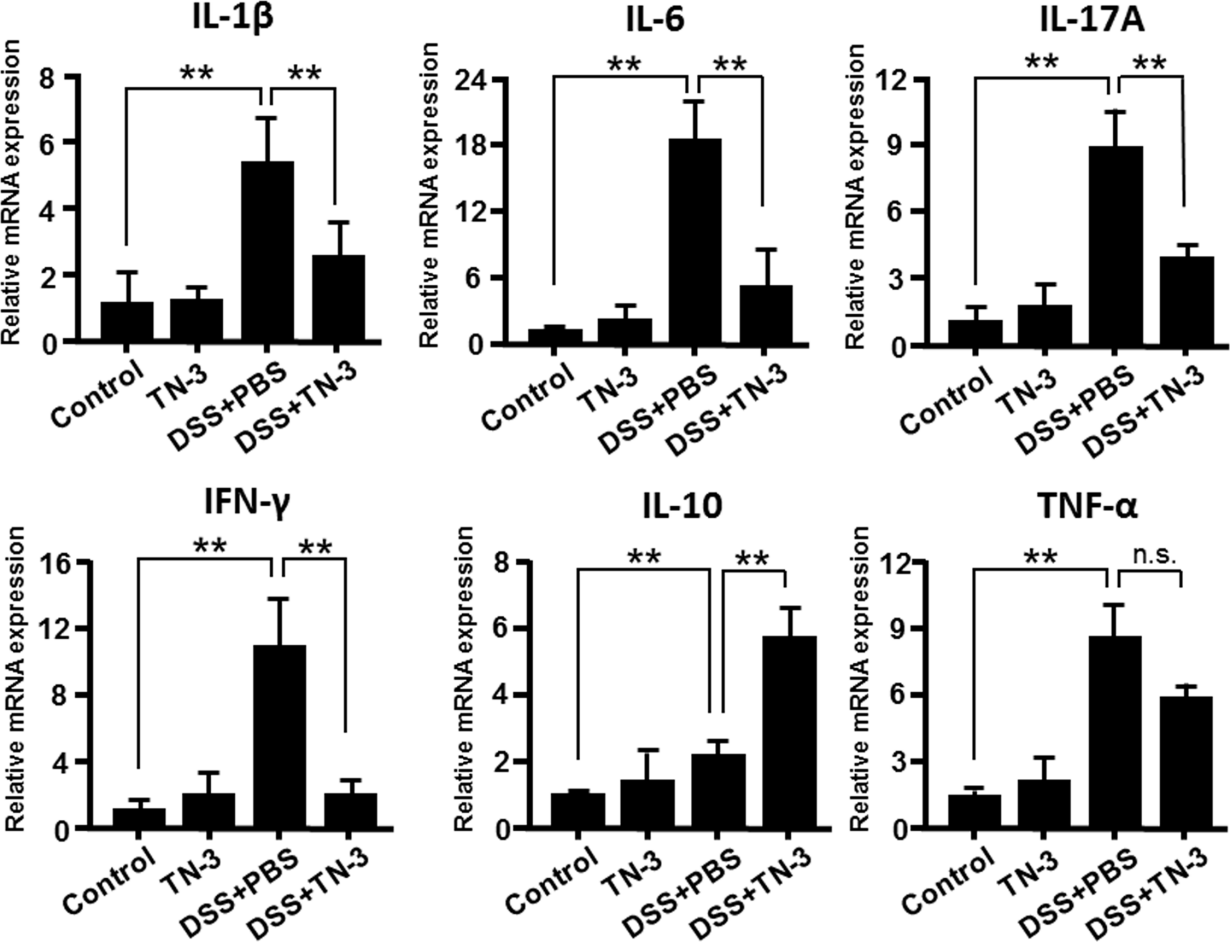
mRNA expression of cytokines in the colon. Real-time PCR analysis for the mRNA expression of cytokines was performed on the colonic mucosa. The cytokine mRNA expression was converted to a value relative to β-actin mRNA expression, and was presented as an increase relative to the results for control mice (no treatment). Data are expressed as means ± SD of five different samples. *P < 0.05, **P < 0.01.

**Fig 5:**
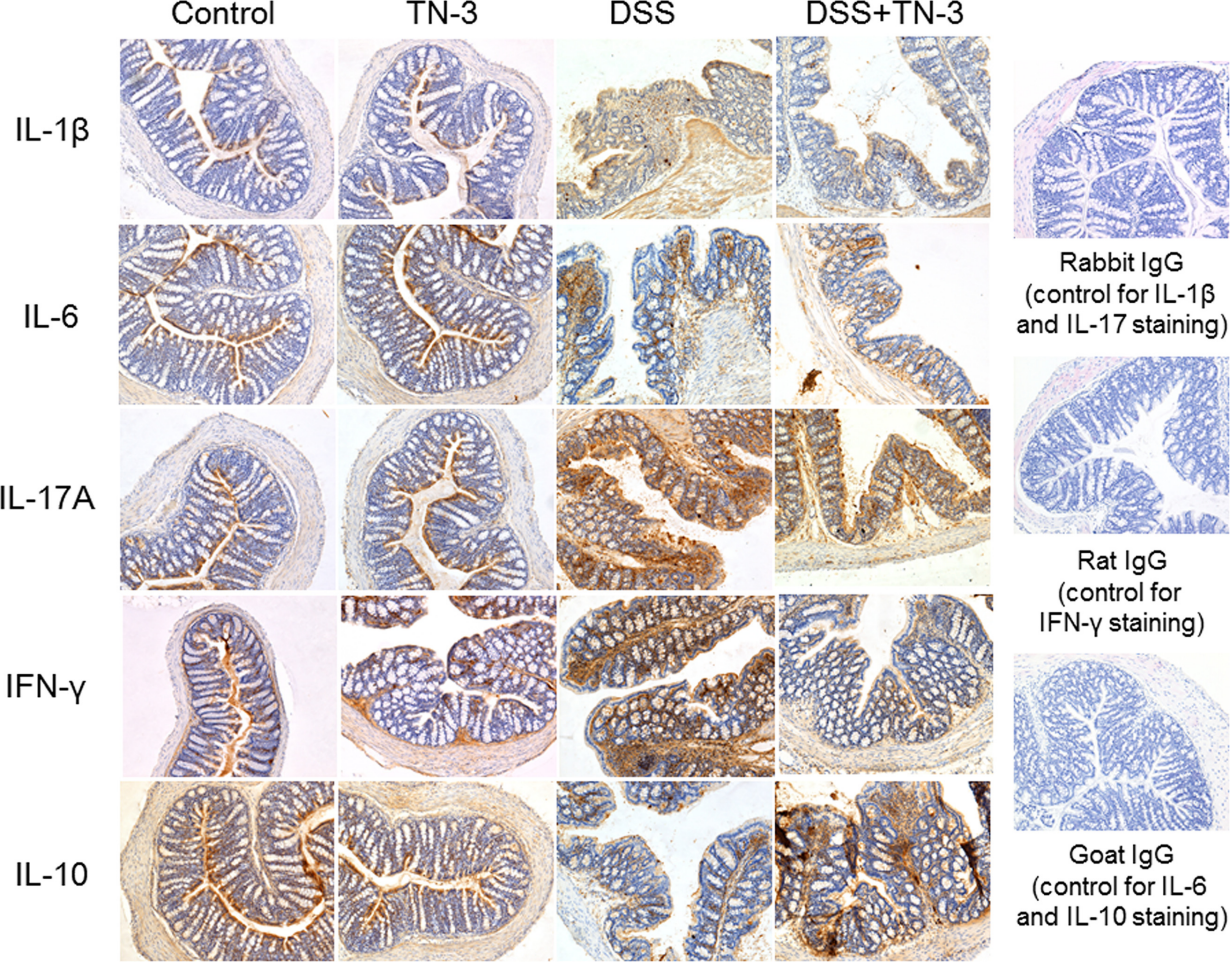
The expression of cytokines in the colon tissue. The expression of IL-1β, IL-6, IL-17A, IFN-γ, and IL-10 in the colon tissues was examined using immunohistochemistry. Control staining was also presented. The data are representative of four independent experiments. Magnification x 100.

The mRNA expression of cytokines in the colonic mucosa was analyzed using real-time PCR. As shown in Fig 4, the mRNA expression of IL-1β, IL-6, IL-17A and IFN-γ decreased significantly in the DSS plus TN-3 mice as compared to the DSS mice. The mRNA expression of TNF-α decreased in the DSS plus TN-3 mice as compared to the DSS mice, but there was no significance between these two groups. Interestingly, we found that the mRNA expression of IL-10, which is an anti-inflammatory cytokine, was significantly elevated in the DSS plus TN-3 mice as compared to the DSS mice. As shown in Fig 5, we also confirmed that the expression of IL-1β, IL-6, IL-17A, and IFN-γ decreased, and the expression of IL-10 increased in the colon tissues of the DSS plus TN-3 mice as compared to the DSS mice using immunohistochemistry. Thus, TN-3 significantly suppressed the expression of proinflammatory cytokines and enhanced IL-10 expression.

### Effect of TN-3 on the induction of regulatory T cells (Treg cells)

**Fig 6:**
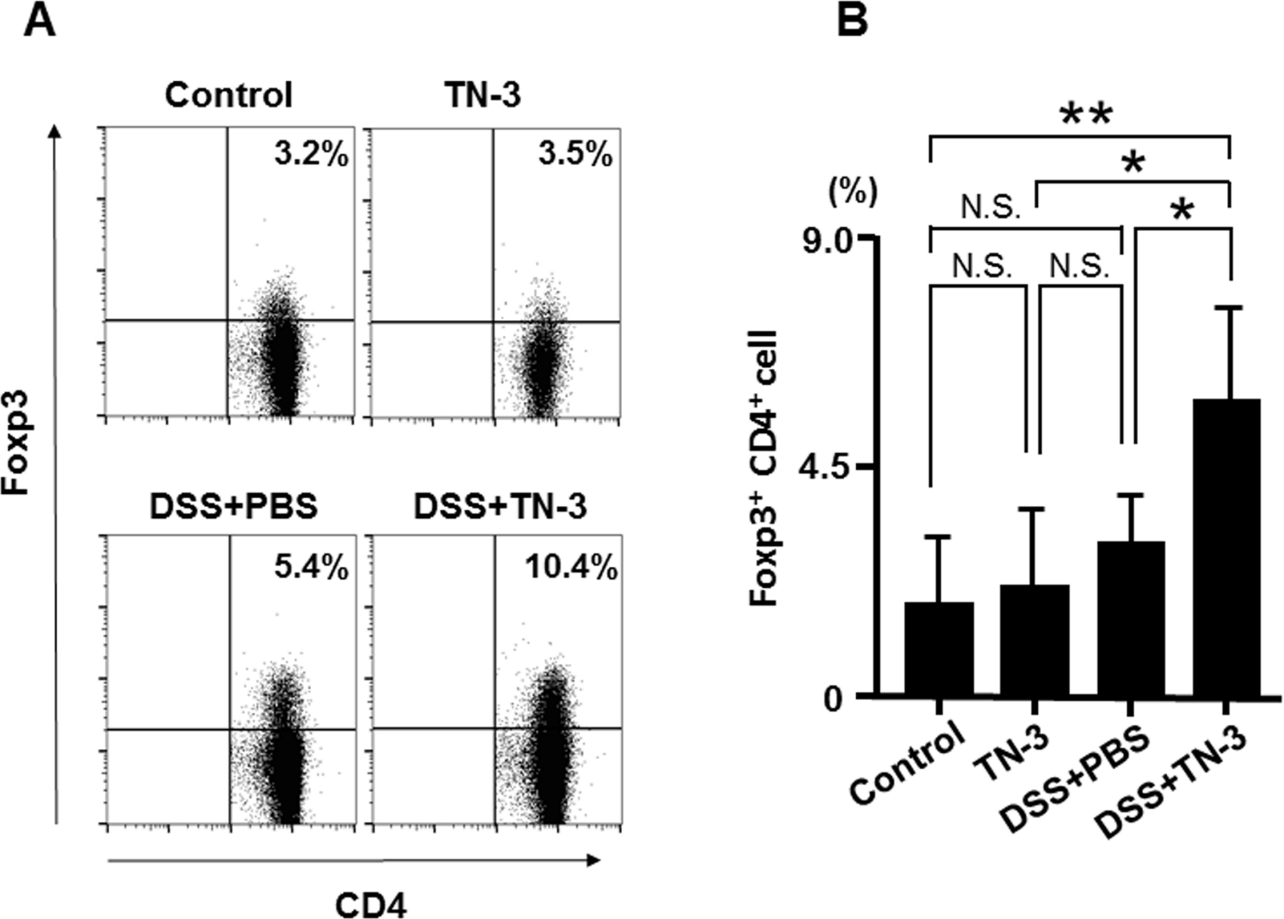
Proportion of Treg cells in the lamina propria of the colon. (A) Flow cytometric analysis for Foxp3^+^CD4^+^ T reg cells in the colonic lamina propria. Data are representative of five independent experiments. (B) Proportion of Foxp3^+^CD4^+^ T reg cells. Data are expressed as means ± SD of five different samples. *P < 0.05, **P < 0.01, n.s.; not significant.

To explore the mechanism underlying the effects of TN-3 on DSS colitis, we focused on Treg cells in the colonic mucosa. Treg cells are known as a major source of IL-10 and have anti-inflammatory effects on the development of colitis [28]. As shown in Fig 6A, Treg cells were detected as CD4^+^Foxp3^+^ double positive cells by flow cytometry. As shown in Fig 6B, the proportion of Treg cells was not altered in the TN-3 or DSS mice as compared to the control mice, but significantly increased in the DSS plus TN-3 mice as compared to the DSS mice. These results suggest that the preventive effects of TN-3 on the development of DSS colitis might be mediated by the induction of Treg cells in the colonic mucosa.

### Effect of TN-3 on fecal short-chain fatty acid (SCFA) levels

**Fig 7:**
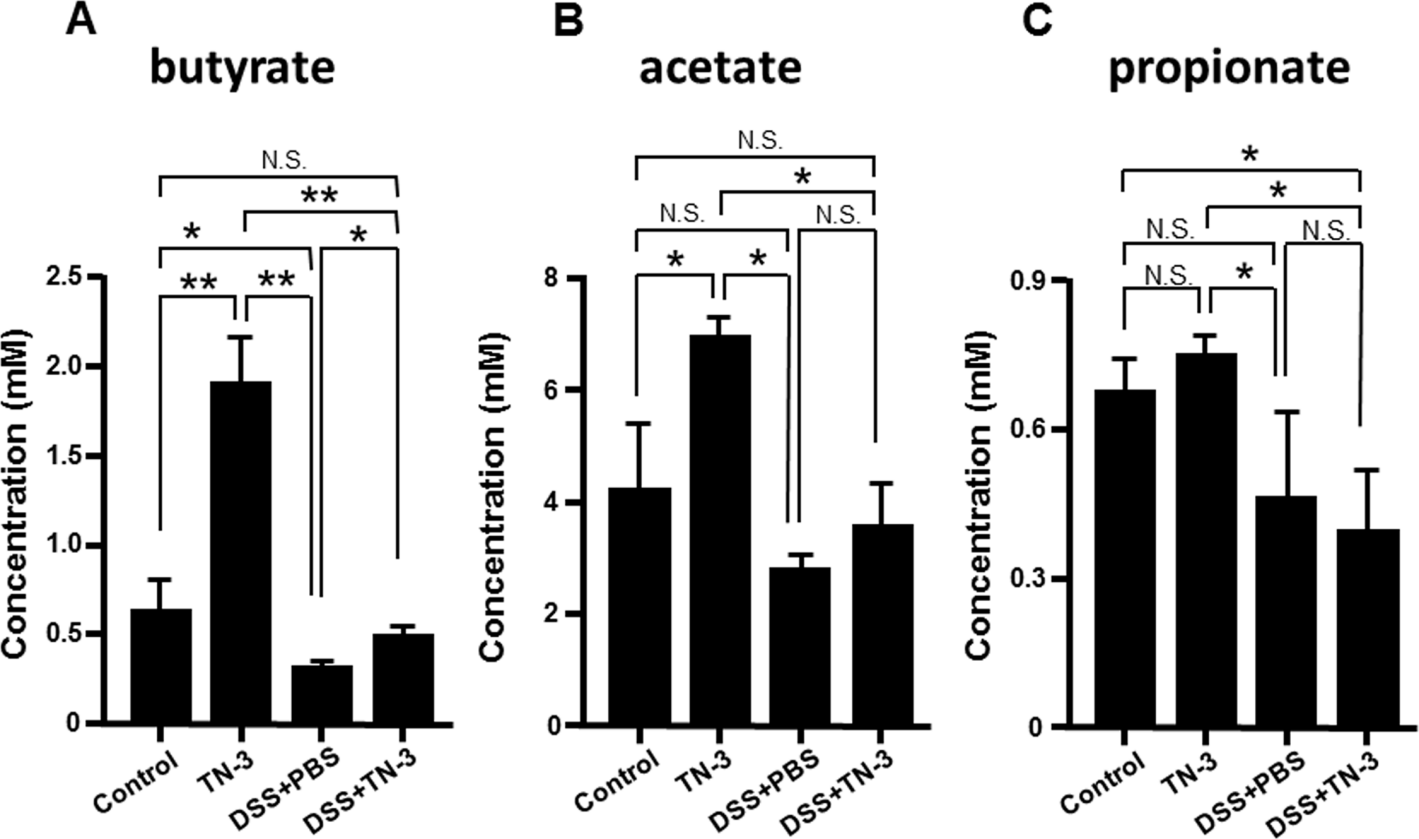
Effect of TN-3 on the concentration of fecal SCFAs. The concentrations of fecal SCFAs were measured by High-performance liquid chromatography: (A) the concentration of butyrate, (B) the concentration of acetate, (C) the concentration of propionate. Data are expressed as means ± SD of five different samples. *P < 0.05, **P < 0.01, n.s.; not significant.

Short-chain fatty acids (SCFAs), such as butyrate, acetate and propionate, are generated by the fermentation of dietary fibers by anaerobic bacteria [29]. Recent studies reported that butyrate plays a crucial role in the induction of mucosal Treg cells [30, 31]. Therefore, we examined the effects of TN-3 on fecal SCFA levels. As shown in Fig 7, fecal butyrate and acetate levels significantly increased in the TN-3 mice as compared to the control mice. Fecal butyrate levels significantly decreased in the DSS mice as compared to the control mice, but increased significantly in the DSS plus TN-3 mice as compared to the DSS mice. On the other hand, acetate levels significantly increased in the TN-3 mice as compared to the control mice, but there was no difference between the DSS and the DSS plus TN-3 mice. We also measured the fecal propionate levels, but there was no significant difference between the TN-3 mice and the control mice, and between the DSS and the DSS plus TN-3.

### Effects of TN-3 on the fecal microbial composition and diversity

**Fig 8:**
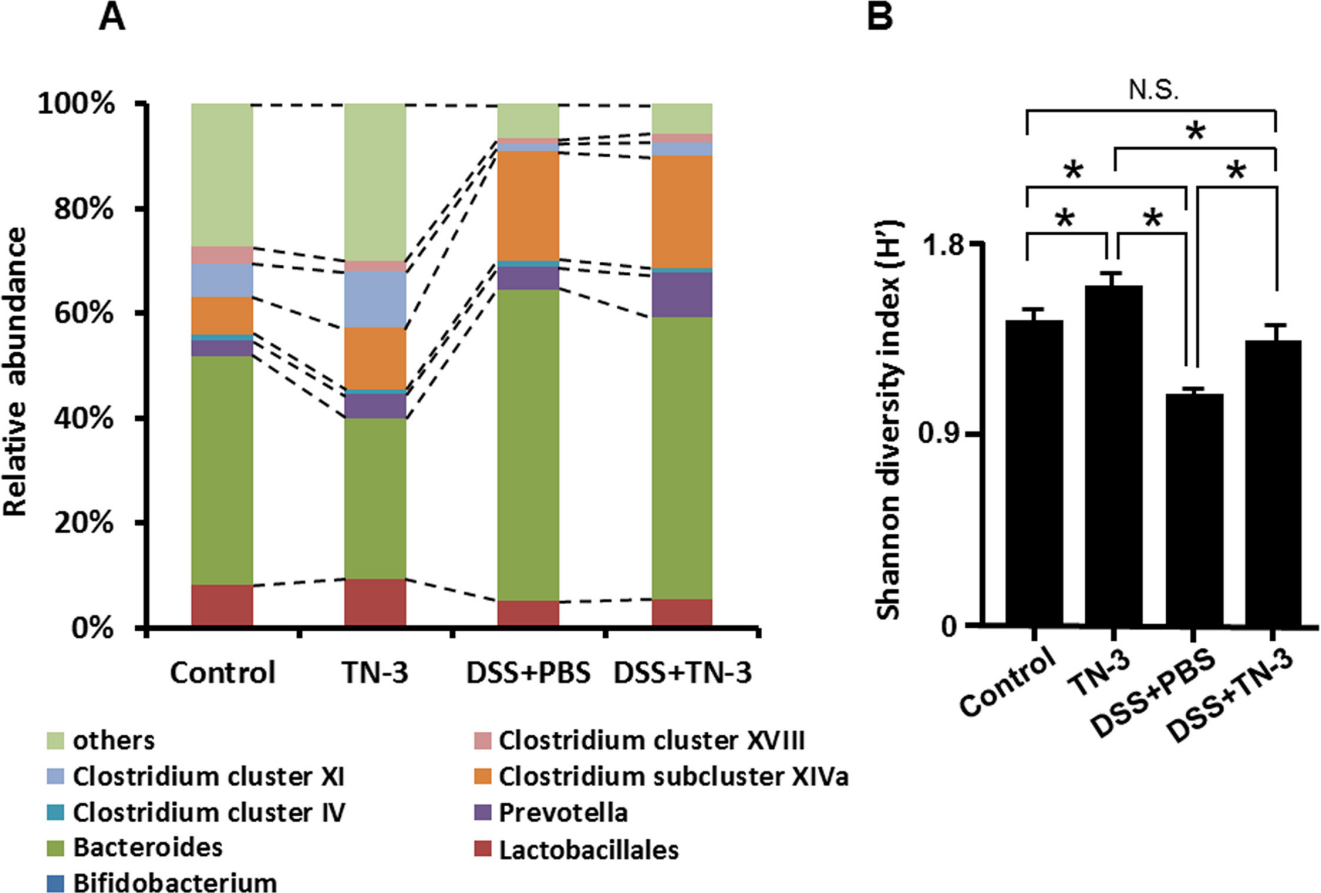
Effects of *E*. *durans* TN-3 on the gut microbial community and its diversity. (A) Changes in the fecal bacteria in DSS- and TN-3-treated mice. The value indicates the percentage of the predicted bacteria. (B) The Shannon Diversity Index (SDI) in comparison of the fecal bacterial diversity between groups. SDI was calculated from the *Bsl*I-digested terminal restriction fragment patterns. Data are expressed as means ± SD of five different samples.

We investigated the effects of TN-3 on the fecal microbial structure using the T-RFLP method. The prediction of bacteria was performed according to the *Bsl*I-digested T-RFLP database [25]. As shown in Fig 8A and Table 2, the results of bacteria prediction by T-RFLP analysis showed that proportion of *Bacteroides* significantly decreased and that of *Clostridium* cluster XI significantly increased in the TN-3 mice as compared to the control mice. In the DSS mice, the proportion of *Bacteroides* and *Clostridium* subcluster XIVa significantly increased and that of *Clostridium* cluster XI significantly decreased as compared to the control mice. However, there was no significant difference in the fecal microbial structure between the DSS mice and the DSS plus TN-3 mice. Next, we calculated the Shannon diversity index in each group. As shown in Fig 8B, the microbial diversity increased significantly in the TN-3 mice but decreased significantly in the DSS mice as compared to the control mice. However, the microbial diversity significantly increased in the DSS plus TN-3 mice as compared to the DSS mice. There was no significant difference in the diversity of microbial community between the control mice and the DSS plus TN-3 mice.

**Table 2:** Comparison of predicted bacteria.

Predicted bacteria	control	TN-3	DSS+PBS	DSS+TN-3
*Bifidobacteriales*	0.2 ± 0.4	0.0	0.0	0.0
*Lactobacillales *	8.1 ± 2.6	9.4 ± 6.1	5.0 ± 3.9	5.6 ± 2.2
*Bacteroides*	43.6 ± 9.4 ^a^	30.6 ± 6.5 ^b^	59.0 ± 8.3 ^c^	52.5 ± 6.0^ac^
*Prevotella*	3.0 ± 1.2	4.6 ± 1.2	4.0 ± 3.3	7.3 ± 4.3
*Clostridium*	18.0 ± 3.5 ^a^	25.4 ± 1.6 ^b^	25.9 ± 6.6 ^ab^	27.0 ± 3.2 ^b^
*Clostridium* cluster IV	1.0 ± 0.61	0.9 ± 0.33	1.1 ± 0.33	0.8 ± 0.35
*Clostridium* subcluster XIVa	7.3 ± 2.06 ^a^	11.8 ± 3.9 ^a^	22.5 ± 6.1 ^b^	21.5 ± 3.4 ^b^
*Clostridium* cluster XI	6.3 ± 1.4 ^a^	10.9 ± 2.7 ^b^	1.3 ± 0.9 ^c^	2.5 ± 1.3 ^c^
*Clostridium* cluster XVIII	3.4 ± 1.5 ^a^	1.8 ± 1.1 ^ab^	1.0 ± 0.27 ^b^	2.2 ± 1.1 ^ab^
Others	27.2 ± 8.1 ^a^	30.1 ± 6.3 ^a^	6.1 ± 3.6 ^b^	11.0 ± 11.0^b^

Each value indicates the percentage of individual predicted bacteria. Values were expressed as mean ± SD. Values not sharing a letter are significantly different.

## Discussion

IBD is heterogeneous diseases characterized by overly aggressive immune responses to a subset of gut bacteria in genetically susceptible individual [3]. The relative imbalance of aggressive and protective bacterial species, termed dysbiosis, has been reported to be one of critical factors involved in the pathogenesis of IBD [4, 32]. Recent studies suggest that the therapeutic approaches targeting the gut microbiota, such as the use of probiotics, prebiotics and synbiotics, may improve the clinical outcome of patients with IBD [3]. The efficacy of probiotics for IBD has been reported. There are some large clinical trials of probiotics in IBD, especially in UC, in the setting of remission and maintenance of remission. A clinical traial demonstrated that *E*. *coli* Nissle 1917 was similar in efficacy to mesalamine for maintaining UC in remission [33]. *E*. *coli* Nissle 1917 is considered an effective alternative to mesalazine for maintenance of remission in UC. Two clinical trials suggested that the use of multistrain probiotic VSL#3 for moderate active UC was able to improve the remission rate and the clinical response rate [34, 35].

Most probiotic microorganisms are classified as lactic acid bacteria, such as *Lactobacillus* spp., *Bifidobacterium* spp. and *Enterococcus* spp.[9] *Enterococcus* spp. strain TN-3 was isolated from deep seawater in Toyama bay in Japan [12]. TN-3 causes liquefaction of gelatin, fermentation of litmus milk, and possesses β-galactosidase activity. TN-3 has high homology to *E*. *durans* with respect to its 16S rDNA nucleotide sequences [12]. Irit Raz *et al*. suggested *E*. *durans* has the immunoprotective and anti-inflammatory effect in DSS-induced colitis. They isolated *E*. *durans*, which was able to produce butyrate, from human feces, and demonstrated that *E*. *durans* suppressed DSS-induced colitis by colonizing in the colon and supplying adequate butyrate to colonocytes [36]. However, the mechanisms of suppressive effect of *E*. *durans* remained unclear. In the current study, we showed that *E*. *durans* TN-3 suppresses the development of DSS colitis via the induction of mucosal Treg cells. This was accompanied by the restoration of the fecal microbial diversity and an increase in fecal butyrate levels.

A number of studies have demonstrated that IL-10 is a key immunomodulatory factor that inhibits the release of proinflammatory cytokines by immune and inflammatory cells [37, 38], and that Treg cells are considered as a major source of IL-10 in the intestinal mucosa [39, 40]. In this study, we found that both IL-10 mRNA expression and the proportion of Treg cells increased significantly in the DSS plus TN-3 mice as compared to the DSS mice. These results suggest that *E*. *durans* TN-3 might induce Treg cells and stimulate IL-10 production, leading to the suppression of the development of DSS colitis.

Recent studies demonstrated that butyrate, generated by the fermentation of dietary fiber by anaerobes, plays a crucial role in the induction of Treg cells in the mucosa [32, 41]. Other studies have demonstrated that butyrate has an anti-inflammatory property by suppressing the activation of transcription factor NF-κB, which is a central transcription factor mediating various inflammatory responses [42, 43]. Based on these findings, we hypothesized that TN-3 modulated the gut microbial community and stimulated butyrate production, leading to the induction of Treg cells. As we expected, the diversity of fecal microbial community was restored in the TN-3 plus DSS mice as compared to the DSS mice, and fecal butyrate levels were significantly elevated in the TN-3 plus DSS mice as compared to the DSS mice. These findings suggest that *E*. *durans* TN-3 modulated the gut microbial community and stimulated butyrate production. Subsequently, Treg cells were induced in response to increased butyrate generation, leading to the suppression of the development of DSS colitis. Additionally, butyrate might directly blocked the development of DSS colitis via its inhibitory action on NF-κB activation.

There are some limitations in this study. First, we did not clearly demonstrate how TN-3 modulates the gut microbiota. Second, there is no direct evidence that the increase expression of IL-10 produced by Treg cells is critical for the improvement of DSS-induced colitis. In the future, further examinations are needed to clarify these limitations.

In conclusion, the preventive administration of *E*.*durans* TN-3 suppressed the development of DSS colitis via the induction of IL-10 producing Treg cells by restoring of the diversity of gut microbiota. These findings suggest that *E*. *durans* TN-3 is a new probiotic candidate for the treatment of IBD.

## Supporting Information

S1 Fig The effect of therapeutic treatment of TN-3 on the development of colitis. BALB/cAJcl mice were orally inoculated with *E*. *durans* TN-3 (10mg/day) at the same time with the start of 4% DSS treatment. The mice were sacrificed at day12 for the experiments. (A) Changes in body weight. (B) Disease activity index on day 12. Data are expressed as means ± SD (n = 4 mice/group). n.s.; not significant.
(TIF)
