# Supplementary figures and images for: Enterococcus durans TN-3 Induces Regulatory T Cells and Suppresses the Development of Dextran Sulfate Sodium (DSS)-Induced Experimental Colitis
Source: PLoS One. 2016 Jul 20;11(7):e0159705. doi: 10.1371/journal.pone.0159705 (PMC4954729; doi:10.1371/journal.pone.0159705)

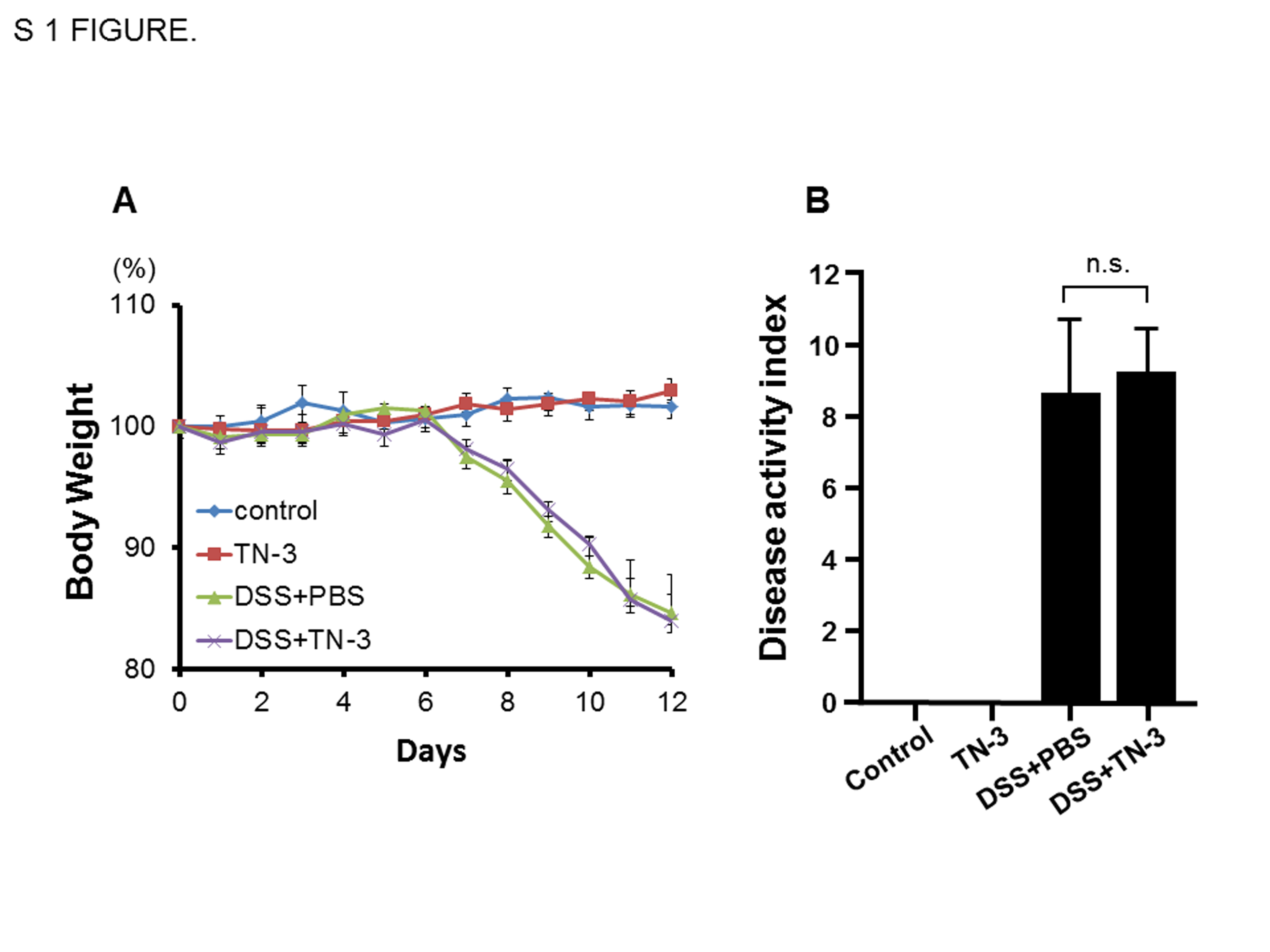

Supplement: S1 Fig — BALB/cAJcl mice were orally inoculated with E. durans TN-3 (10mg/day) at the same time with the start of 4% DSS treatment. The mice were sacrificed at day12 for the experiments. (A) Changes in body weight. (B) Disease activity index on day 12. Data are expressed as means ± SD (n = 4 mice/group). n.s.; not significant. (TIF) [file pone.0159705.s001.tif]
